# Young Adult Utilization of a Smoking Cessation Website: An Observational Study Comparing Young and Older Adult Patterns of Use

**DOI:** 10.2196/resprot.4881

**Published:** 2016-07-11

**Authors:** Jennifer Cantrell, Vinu Ilakkuvan, Amanda L Graham, Amanda Richardson, Haijun Xiao, Robin J Mermelstein, Susan J Curry, Amy K Sporer, Donna M Vallone

**Affiliations:** ^1^ Evaluation Science and Research Truth Initiative Washington, DC United States; ^2^ Department of Health, Behavior and Society Johns Hopkins Bloomberg School of Public Health Baltimore, MD United States; ^3^ Department of Prevention and Community Health George Washington University Milken Institute School of Public Health Washington, DC United States; ^4^ The Schroeder Institute for Tobacco Research and Policy Studies Truth Initiative Washington, DC United States; ^5^ Department of Oncology Georgetown University Medical Center/Cancer Prevention and Control Program Lombardi Comprehensive Cancer Center Washington, DC United States; ^6^ Department of Health Behavior University of North Carolina Gillings School of Global Public Health Chapel Hill, NC United States; ^7^ Institute for Health Research and Policy University of Illinois at Chicago Chicago, IL United States; ^8^ Department of Psychology University of Illinois at Chicago Chicago, IL United States; ^9^ College of Public Health University of Iowa Iowa City, IA United States; ^10^ College of Global Public Health New York University New York, NY United States

**Keywords:** young adults, smoking cessation, Internet, utilization

## Abstract

**Background:**

There is little research on how young adults or young adult subgroups utilize and engage with Web-based cessation interventions when trying to quit smoking. Addressing this knowledge gap is important to identify opportunities to optimize the effectiveness of online cessation programs across diverse young adult users.

**Objective:**

This study examines utilization of the BecomeAnEX.org smoking cessation website among young adults and young adult subgroups compared with older adults to identify patterns of use by age, gender, and race/ethnicity.

**Methods:**

Study participants were 5983 new registered users on a free smoking cessation website who were aged 18 to 70 years. Website utilization was tracked for 6 months; metrics of use included website visits, pages per visit, length of visit, and interaction with specific website features. Differences in website use by age were examined via bivariate analyses and multivariate logistic regression adjusted for age, gender, and race/ethnicity. Interactions were examined to determine differences by gender and race/ethnicity within young (18- to 24-year-olds and 25- to 34-year-olds) and older (35 years and older) adult segments.

**Results:**

A greater percentage of young adults aged 18 to 34 years visited the site only once compared with older adults aged 35 years and older (72.05% vs 56.59%, respectively; *P*<.001). Young adults also spent less time on the site and viewed fewer pages than older adults. In adjusted analyses, young adults were significantly less likely than older adults to visit the site more than once (18-24 years: adjusted odds ratio [AOR] 0.58, 95% CI 0.49-0.68, *P*<.001; 25-34 years: AOR 0.56, 95% CI 0.50-0.64, *P*<.001), spend more than 3 minutes on the site (18-24 years: AOR 0.67, 95% CI 0.57-0.79, *P*<.001; 25-34 years: AOR 0.56, 95% CI 0.49-0.64, *P*<.001), view 12 or more pages (18-24 years: AOR 0.72, 95% CI 0.61-0.83; *P*<.001; 25-34 years: AOR 0.67, 95% CI 0.59-0.76, *P<*.001), utilize the BecomeAnEX.org community (18-24 years: AOR 0.61, 95% CI 0.48-0.79, *P*<.001; 25-34 years: AOR 0.73, 95% CI 0.60-0.88, *P*<.001), or utilize Separation Exercises (18-24 years: AOR 0.68, 95% CI 0.51-0.89, *P*<.01; 25-34 years: AOR 0.77, 95% CI 0.63-0.94, *P*<.01). Gender differences in utilization were more pronounced among young adults compared with older adults, with lower levels of utilization among young men than young women. For all age groups, utilization was higher among whites and African Americans than among Hispanics and other racial minorities, with one exception—BecomeAnEX.org community utilization was significantly higher among Hispanic young adults compared with white and African American young adults.

**Conclusions:**

Results point to important areas of inquiry for future research and development efforts. Research should focus on enhancing demand and increasing engagement among younger adults and men, examining strategies for capitalizing on young adult developmental needs, and increasing utilization of effective site features among diverse young adult users.

## Introduction

Tobacco smoking remains the leading cause of preventable disease and death in the United States, with 16.8% of US adults (40 million individuals) reporting current cigarette smoking in 2014 [1,2]. The adult smoking rate has declined significantly in the last decade from 20.9% in 2005 to 16.8% in 2014 [2], and further reductions require continued comprehensive tobacco control efforts of which cessation interventions are a key component [3,4]. Helping young adult smokers quit in particular is critical to ensure continued reductions in population-level cessation.

Young adulthood is a critical developmental period for tobacco use and cessation. Substance use–related risk behavior peaks during young adulthood [5]. As individuals age into this time of life, they gain the ability to purchase tobacco legally in the United States and may enter a phase of transition from experimental to regular tobacco use [6]. Smoking often becomes entrenched during these years, and young adults are increasingly a prime target for tobacco marketing [7,8]. Critically, research indicates quitting before age 35 can result in a life expectancy on par with that of never smokers [9].

Young adults are more likely than older adults to make a quit attempt [10] but less likely to use assistance when trying to quit [10,11]. Curry et al [10] analyzed data from the 2005 National Health Interview Survey to examine use of evidence-based treatment by young adults (18-24 years) compared to older adults (25 years and older). Young adults reported very low levels of cessation treatment with only 4% using any behavioral treatment (eg, telephone quit line, class/clinic/group, one-on-one counseling). Young adults were also much less likely to report using any pharmacotherapy (eg, nicotine replacement therapy, bupropion) as compared with older adults (18% vs 33%, respectively). Less than 5% of these young adults reported using other types of cessation such as printed self-help materials, Internet, or hypnosis/acupuncture [10].

Comprehensive reviews have documented promising evidence for the efficacy of online interventions for smoking cessation [12-28]. Web-based interventions can promote cessation among adults, particularly if they are tailored to individuals and maximize interactivity to engage users [12,14]. Given the increasing utilization of the Internet among US adults, online interventions have the potential for substantial reach and population-level impact in reducing smoking [4]. A majority of adults across all age, race/ethnicity, income, and educational groups are Internet users [30]. The greatest increases in Internet adoption in recent years have occurred among those with the lowest rates of cessation [30,31], suggesting the Internet may be an increasingly feasible channel for intervention among these groups. With users of all ages having expanded access to the Internet, online interventions become more convenient [14]. Further, low costs per user make online interventions cost effective given sufficient reach [14,15].

While Web interventions may be important for reaching adults across all age groups, this channel may be especially relevant for reaching younger smokers. Young adults aged 18 to 29 years have demonstrated consistently higher levels of Internet adoption than older groups in the past 15 years and currently report Internet use at a nearly ubiquitous rate of 96% [30]. Young adults also use social networking sites at high rates, with 89% of young adult Internet users reporting social network site use in contrast to only 74% of adult Internet users overall [32].

Further, young adult smokers have indicated interest in using technology-based interventions [33], and Web-based resources have been identified as a potentially promising intervention for younger smokers [27,28,34-39]. Although An et al [40] reported very low levels of participation in a Web-based cessation intervention among college students during the beta phase of development, utilization increased sharply as the site was changed from a stop-smoking website to an online college life magazine and proactive peer email support and directed activities were added. Age did not moderate treatment effects in the formal outcome evaluation [36]. In a 3-group randomized trial, An et al [27] found high rates of 30-day abstinence and intervention engagement among 18- to 30-year-old smokers in a personally tailored online intervention that utilized an avatar host for individual personal health “makeovers” plus video-based online peer coaching. An additional study by Berg et al [4] found higher rates of intervention engagement and fewer cigarettes smoked per day among participants randomized to an intervention with online health behavior monitoring and targeted messages compared with those randomized to the online American Cancer Society Guide to Quitting Smoking. In one of the few studies of an open-access smoking cessation website, Richardson et al [17] found no differences between young and older adults on overall number of visits to the website or use of the community forum but did find that young adults were more likely to access an interactive feature about managing smoking triggers. Together, these studies along with research on media utilization demonstrate that young adults are extensive users of digital and social media and suggest that online interventions may play an important role in reaching and engaging a young adult audience.

There is little research focused on examining patterns of engagement on cessation websites among specific populations. While metrics used to capture engagement differ somewhat across studies, common metrics generally include some measures of website “dose” to capture potential exposure to or use of the content of the website intervention [41-43]. Such metrics commonly include number of visits or log-ins [16,17,19-21,23,26,28,43-46], average session length in minutes and/or total minutes on the site [16,17,23,28,43], and total number of pages viewed [16,17,23,28] or Web sections opened or read [16,18,47]. Other engagement metrics capture utilization of specific website components, including use of interactive tools such as setting a quit date [17,43,47], identifying triggers [17,43], tracking or monitoring one’s smoking behaviors [17,28,40,47], and watching videos [27,28,43]. Additional program components often captured include use of or participation in an online community in some form, which may involve accessing the community and/or reading posts [16,17,36,43], posting messages [16,23,36,43,48], and sending or receiving personal messages [23,36,43,45].

Only a few of these studies provide details on how users engage with website interventions or examine utilization outside of a controlled trial setting [17,23,26,36,45,46]. Fewer still focus on young adults or young adult subgroups [27,28,39,40]. Understanding how young adult smokers utilize cessation websites in a real-world setting is a necessary first step in optimizing interventions for engagement and ensuring they are effective for youth audiences and diverse groups of users. This observational study was conducted to identify patterns of website engagement among young adults compared with older adults and among subgroups defined by gender and race/ethnicity, with a focus on young adults. This study adds to a relatively small body of literature specifically focused on the role of digital and social media interventions in promoting cessation among young adults.

## Methods

### BecomeAnEX.org Intervention

BecomeAnEX.org is an evidence-based smoking cessation website developed by Truth Initiative (formerly American Legacy Foundation), a national nonprofit organization that, in partnership with the Mayo Clinic’s Nicotine Dependence Center, focuses on smoking prevention and cessation. Launched in 2008, the website was designed for a target audience broadly defined as smokers between the ages of 25 and 49 years who were open to quitting smoking [49]. Promotional activities and the voice of the site were designed to appeal to blue-collar smokers of low to moderate incomes because of their higher smoking rates. The site was developed in accordance with the 2008 US Department of Health and Human Services Public Health Service Clinical Practice Guidelines for Treating Tobacco Dependence (PHS Guideline) [50] and focuses on educating and supporting smokers throughout their quitting efforts. The site is available on multiple platforms, including mobile, and is designed to engage smokers through the following core elements: (1) My Quit Plan, a checklist that helps smokers set a quit date and tracks their progress through the steps of the quit plan; (2) Beat Your Smoking Triggers, a list of common triggers with tips for dissociating cigarettes from them; (3) a Cigarette Tracker to help smokers identify and track their smoking triggers; (4) Separation Exercises to teach smokers how to separate smoking a cigarette from the activities that trigger cravings (ie, drinking coffee or alcohol); (5) a Support Exercise to help smokers identify who they can turn to for support when quitting smoking; (6) Quit Smoking Resources, a list of national organizations, state quitlines, and websites that smokers can turn to for additional information and help to quit smoking; (7) texts and videos detailing the importance of medication and the different types available; and (8) the BecomeAnEx.org community, a large online network of current and former smokers.

BecomeAnEX has been promoted through a national multimedia campaign since 2008 [49]. During the study period, registration on BecomeAnEX.org required users to indicate age, gender, and race/ethnicity. Registration also required users to agree to the site’s Terms of Use and Privacy Policy which state that (1) Truth Initiative collects information about its users and their use of the site, (2) information is used for research and quality improvement purposes only, and (3) personal information is kept confidential. Informed consent was not required given the availability of website utilization data and the observational nature of the study.

### Study Participants

Study participants were 5983 users aged 18 to 70 years who registered on BecomeAnEX.org between May 7, 2012, and November 18, 2012. We excluded new registered users that were recruited to other research studies being conducted within BecomeAnEX at the time (n=3800) and those without a valid email address (n=3566).

### Data Collection and Measures

Demographic data (age, gender, race/ethnicity) gathered during site registration were extracted from the BecomeAnEX database. Website utilization data were collected using Adobe Analytics, a customizable Web analytics product. Every page view by a participant is recorded into a relational database, and page views are grouped into sessions. The duration of a session is defined as the time elapsed between the first page view and the last page view in a given session. If a user does not view a new page for over 30 minutes, the system marks them as inactive and their next return visit creates a new session. We focused on 6-month metrics of utilization given prior research that has shown that the majority of utilization occurs during this time frame [51]. Two types of measures here were utilized to capture engagement with the website. Dose measures were based on number of visits to the site, time spent on the site (minutes), and total number of pages viewed. Engagement metrics to capture utilization of program components included use of the BecomeAnEX.org community and use of Separation Exercises. We chose these two features because each independently predicted abstinence in a previous evaluation [17]. We calculated total time spent on the site over the 6-month observation period as the product of minutes per visit and number of visits.

### Analysis

For regression analyses, young adults were categorized into two age groups commonly used to examine tobacco use and cessation for this population: 18 to 24 years and 25 to 34 years [52-55]. Older adults were categorized as aged 35 years and older. For descriptive analyses, 18- to 24-year-olds and 25- to 34-year-olds were combined for succinctness of presentation and compared with older adults. Race and ethnicity were categorized as non-Hispanic white, non-Hispanic African American, Hispanic, and other non-Hispanic.

Descriptive analyses were used to examine differences in site utilization patterns between young adults (18-34 years) and older adults (35 years and older). Key variables of interest included number of site visits, time spent on the site in minutes, and number of page views; use of the BecomeAnEX.org community forum and Separation Exercises were examined as binary variables (none vs any). In addition to examining utilization metrics as continuous data, we created categorical variables based on the meta-analysis presented in the PHS Guideline [50] to provide a framework for interpreting the intensity of website utilization. Using the PHS Guideline for number of sessions, we categorized number of site visits as 1, 2-3, 4-8, and more than 8 visits. Using the PHS Guideline categories for total amount of contact time, we categorized time spent on the site as 3 minutes or less, 4 to 30 minutes, and 31 or more minutes. To test differences in utilization metrics by age group, we used the Wilcoxon rank-sum test for nonnormally distributed continuous variables and the chi-square test for categorical variables.

Main effects logistic or linear regressions were conducted with each of the five utilization metrics, with age group (18-24 years, 25-34 years, and 35 years and older) as the main predictor and gender and race/ethnicity as controls. For purposes of regression analyses, number of site visits was dichotomized as 1 visit (registration visit only) versus 2 or more visits; time on site was dichotomized as 3 minutes or less versus more than 3 minutes. Total pages viewed was dichotomized at the median (11 pages or fewer vs 12 or more pages), while website feature use was dichotomized as never versus any use. Given the large sample size and number of dependent measures examined (n=5), we used a criterion of *P*<.01 (.05/5) as a more conservative gauge of statistical significance.

We also conducted regressions for each outcome with age, gender, and race/ethnicity and two interactions by age and gender as well as age and race/ethnicity, with interactions considered significant at the *P*<.05 level. Models were then stratified by the three age groups and rerun.

## Results

### Sample Description

Young adults aged 18 to 34 years comprised 50.64% (3030/5983) of the overall sample (see Table 1). The average age of the sample overall was 37.3 years (standard error [SE] 0.16) with an average age of 21.8 years (SE 0.06) for 18- to 24-year-olds, 29.3 years (SE 0.06) for 25- to 34-year-olds, and 48.0 (SE 0.16) for older adults (see Multimedia Appendix 1). Age groups were 18 to 24 years, 16.00% (957/5983); 25 to 34 years, 34.60% (2073/5983); 31 to 49 years, 40.82% (2442/5983); and 50 years and older, 21.39% (1280/5983). The gender distribution was more evenly split for 18- to 34-year-old young adults than for adults over 35 years (54.00% [1567/3030] female and 69.02% [1998/2953] female, respectively). Among young adults, whites comprised the majority of users; however, there were fewer non-Hispanic whites and a greater proportion of Hispanics and other non-Hispanics compared with older adults. See Multimedia Appendix 1 for further breakdown of demographics for young adults aged 18 to 24 years and 25 to 34 years.

**Table 1:** Participant Characteristics.

		Young adults 18-34 years n=3030	Older adults 35 years and older n=2953	Full sample n=5983
Age, years, mean (SE)		26.9 (0.08)	48.0 (0.16)	37.3 (0.16)
**Gender, n (%)**				
	Female	1567 (54.00)	1998 (69.02)	3565 (61.50)
	Male	1335 (46.00)	897 (30.98)	2232 (38.50)
**Race/ethnicity, n (%)**				
	White, non-Hispanic	1753 (57.85)	2268 (76.80)	4021 (67.21)
	African American, non-Hispanic	205 (6.77)	261 (8.84)	466 (7.79)
	Hispanic	591 (19.50)	208 (7.04)	799 (13.35)
	Other, non-Hispanic	481 (15.87)	216 (7.31)	697 (11.65)

### Website Utilization Differences by Age: Bivariate Analyses

The mean number of visits to the website was 5.02 (SE 0.70) overall, with a significant difference in mean visits by age (2.52 [SE 0.15] for adults aged 18 to 34 years and 7.59 [SE 1.41] for older adults; *P*<.001) (see Table 2). While a majority of both younger and older adults visited the site only once, the proportion of individuals who visited the site only once versus two times or more differed significantly by age, with 72.05% (2183/3030) of younger adults accessing the site only once compared with 56.59% (1671/2953) of older adults (*P*<.001). Younger adults also spent significantly less time on the site compared with older adults. Overall, the mean number of pages viewed was 77.11 (SE 11.98), with older adults viewing more than 2.5 times as many pages as younger adults (113.01 [SE 23.61] vs 42.00 [SE 5.44], respectively; *P*<.001). Younger adults were also significantly less likely to use the BecomeAnEX.org community and Separation Exercises features compared with older adults *.* Multimedia Appendix 1 shows bivariate analyses of website characteristics for each young adult group and older adults and comparisons for each group. Significant differences in utilization for the 18- to 24-year-old and 25- to 34-year-old subgroups in comparison with the older adults were similar to those described above. There were no significant differences in utilization between the 18- to 24-year-olds versus 25- to 34-year-olds, with the exception of mean time on site (28.50 minutes [SE 3.91] vs 27.12 minutes [SE 3.04] for 18- to 24-year-olds and 25- to 34-year-olds, respectively) and pages viewed (67.50 pages [SE 15.63] vs 30.41 pages [SE 3.32] for 18- to 24-year-olds and 25- to 34-year-olds, respectively).

**Table 2:** Bivariate analyses of website utilization metrics by age.

			Young adults 18-34 years n=3030	Older adults 35 years and older n=2953	Full sample	*P* value
**Site visits**						
	Mean (SE^a^)		2.52 (0.15)	7.59 (1.41)	5.02 (0.70)	<.001
	**Median (IQR**^b^)		1.00 (1.00)	1.00 (2.00)	1.00 (1.00)	
		1 time, n (%)	2183 (72.05)	1671 (56.59)	3854 (64.42)	
		2-3 times, n (%)	527 (17.39)	758 (25.67)	1285 (21.48)	
		4-8 times, n (%)	211 (6.96)	341 (11.55)	552 (9.23)	
		>8 times, n (%)	109 (3.60)	183 (6.20)	292 (4.88)	
**Time on site, minutes**						
	Mean (SE)		27.56 (2.42)	140.42 (32.84)	83.26 (16.27)	<.001
	**Median (IQR)**		5.07 (16.27)	10.98 (26.86)	7.30 (21.87)	
		≤3 minutes, n (%)	1232 (40.67)	713 (24.15)	1945 (32.52)	
		4-30 minutes, n (%)	1330 (43.91)	1497 (50.71)	2827 (47.27)	
		≥31 minutes, n (%)	467 (15.42)	742 (25.14)	1209 (20.21)	
**Pages viewed**						
	Mean (SE)		42.00 (5.44)	113.01 (23.61)	77.11 (11.98)	<.001
	Median (IQR)		8.00 (17.00)	14.00 (26.00)	11.00 (22.00)	
Used EX Community, n (%)			327 (10.79)	387 (13.11)	714 (11.93)	.006
Used Separation Exercises, n (%)			232 (7.66)	366 (12.39)	598 (10.00)	<.001

^a^SE: standard error

^b^IQR: interquartile range

### Website Utilization Differences by Age, Gender, and Race/Ethnicity: Multivariate Analyses

Table 3 shows multivariate regressions for each of the website utilization metrics with age, gender and race/ethnicity as predictor variables. Both 18- to 24-year-olds and 25- to 34-year-olds and men were less likely to visit the website more than once, spend more than 3 minutes on the site, view 12 pages or more, use the BecomeAnEX.org community, or use the Separation Exercises compared with older adults and women. Hispanics were less likely than non-Hispanic whites to visit the website more than once, spend more than 3 minutes on the site, view 12 pages or more, or use the Separation Exercises, but they were twice as likely to use the BecomeAnEX.org community. Analyses indicated lower utilization of the website on all metrics among African Americans, but odds ratios (ORs) only reached significance for visiting the site more than once. Those identifying as other were less likely than non-Hispanic whites to spend more than 3 minutes on the site, view 12 pages or more, or use the Separation Exercises.

**Table 3:** Odds ratios (95% confidence intervals) of multivariate regression models of general website utilization metrics.^a^

		Site Visits^b^	Time on site^c^	Page views^d^	Used Community^e^	Used Separation Exercises^f^
**Age**						
	Older adults: 35+	Referent	Referent	Referent	Referent	Referent
	Young adults: 25-34	0.56 (0.50, 0.64)	0.56 (0.49, 0.64)	0.67 (0.59, 0.76)	0.73 (0.60, 0.88)	0.77 (0.63, 0.94)
	Young adults: 18-24	0.58 (0.49, 0.68)	0.67 (0.57, 0.79)	0.72 (0.61, 0.83)	0.61 (0.48, 0.79)	0.68 (0.51, 0.89)
**Gender**												
	Female	Referent	Referent	Referent	Referent	Referent
	Male	0.74 (0.65, 0.83)	0.44 (0.39, 0.50)	0.52 (0.47, 0.59)	0.69 (0.58, 0.83)	0.51 (0.42, 0.63)
**Race/ethnicity**						
	White, non-Hispanic	Referent	Referent	Referent	Referent	Referent
	African American, non-Hispanic	0.72 (0.58, 0.88)	0.88 (0.71, 1.10)	0.82 (0.67, 0.99)	0.75 (0.53, 1.06)	0.87 (0.64, 1.19)
	Hispanic	0.55 (0.46, 0.67)	0.37 (0.31, 0.44)	0.38 (0.32, 0.45)	2.09 (1.66, 2.63)	0.28 (0.18, 0.44)
	Other, non-Hispanic	0.92 (0.76, 1.11)	0.63 (0.53, 0.76)	0.66 (0.55, 0.79)	1.16 (0.88, 1.54)	0.66 (0.47, 0.92)

^a^A criterion of *P*<.01 was used to determine statistical significance.

^b^Site visits: 1 visit (registration visit) versus 2 or more visits.

^c^Time on site: 3 minutes or less versus more than 3 minutes.

^d^Page views: 11 pages or fewer versus 12 pages or more.

^e^Used Community: never versus any use.

^f^Use Separation Exercises: never versus any use.

### Website Utilization Differences by Gender and Race/Ethnicity: Multivariate Analyses Stratified by Age

Interactions with age and gender were significant in models for all outcomes except Separation Exercises. Table 4 shows regressions stratified by each age group. Among both 18- to 24-year-olds and 25- to 34-year-olds, men were significantly less likely than women to spend more than 3 minutes on the site, view 12 or more pages, use the BecomeAnEX.org community, or use the Separation Exercises. Men aged 25 to 34 years were also less likely to have visited the site more than once. Among older adults, men were also significantly less likely than women to spend more than 3 minutes on the site, view 12 or more pages, or use Separation Exercises. Differences in website utilization between older men and women were not as large as those between younger men and women. There were no differences in visiting the site more than once or use of the BecomeAnEX.org community between older men and women.

Interactions with age and race/ethnicity indicated significant differences for visits, viewing pages, and use of the BecomeAnEX.org community. Stratified models in Table 4 indicate that there were no differences in visits by race among the 18- to 24-year-olds. Among the 25- to 34-year-olds and those 35 years and older, Hispanics were significantly less likely to have visited the site more than once compared with non-Hispanic whites, and among those 35 years and older only, African Americans were less likely to have visited the site more than once compared with non-Hispanic whites. For 18- to 24-year-olds, Hispanics and other non-Hispanics were significantly less likely to spend more than 3 minutes on the site compared with non-Hispanic whites, while among 25- to 34-year-olds and those 35 years and older, only Hispanics were less likely to spend more than 3 minutes or view 12 or more pages compared with non-Hispanic whites. While there were no differences in the use of the BecomeAnEx.org community by race/ethnicity for older adults, 18- to 24-year-old Hispanics were 2.4 times more likely to use the community and 25- to 34-year-old Hispanics were 3.5 times more likely to visit the community compared with their non-Hispanic white counterparts. There were no differences by race/ethnicity in use of the Separation Exercises for 18- to 24-year-olds, but 25- to 34-year-olds and those 35 years and older were less likely to use the Separation Exercises compared with their non-Hispanic white counterparts.

**Table 4:** Odds ratios (95% confidence intervals) of multivariate regression models of website utilization by gender, race/ethnicity, and age.^a^

			Site visits^b^	Time on site^c^	Page views^d^	Used Community^e^	Used Separation Exercises^f^
**Young adults: 18-24 years**							
	**Gender**						
		Female	Referent	Referent	Referent	Referent	Referent
		Male	0.75 (0.56, 1.01)	0.43 (0.33, 0.57)	0.48 (0.36, 0.63)	0.51 (0.31, 0.84)	0.43 (0.25, 0.76)
	**Race/ethnicity**						
		White, non-Hispanic	Referent	Referent	Referent	Referent	Referent
		African American, non-Hispanic	0.76 (0.43, 1.35)	0.68 (0.41, 1.16)	0.61 (0.36, 1.02)	1.10 (0.42, 2.91)	0.44 (0.13 1.44)
		Hispanic	0.97 (0.64, 1.46)	0.53 (0.36, 0.78)	0.64 (0.43, 0.94)	2.42 (1.34, 4.39)	0.56 (0.25, 1.27)
		Other, non-Hispanic	1.00 (0.67, 1.48)	0.46 (0.32, 0.68)	0.54 (0.37, 0.79)	2.34 (1.32, 4.14)	0.48 (0.21, 1.08)
**Young adults: 25-34 years**							
	**Gender**						
		Female	Referent	Referent	Referent	Referent	Referent
		Male	0.56 (0.45, 0.69)	0.30 (0.25, 0.37)	0.36 (0.30, 0.45)	0.45 (0.32, 0.63)	0.38 (0.26, 0.56)
	**Race/ethnicity**						
		White, non-Hispanic	Referent	Referent	Referent	Referent	Referent
		African American, non-Hispanic	0.75 (0.50, 1.13)	0.88 (0.60, 1.31)	0.86 (0.59, 1.24)	0.70 (0.33, 1.48)	1.08 (0.61, 1.92)
		Hispanic	0.55 (0.41, 0.74)	0.32 (0.25, 0.42)	0.36 (0.27, 0.48)	3.49 (2.43, 5.00)	0.21 (0.10, 0.47)
		Other, non-Hispanic	0.92 (0.67, 1.25)	0.74 (0.55, 0.98)	0.75 (0.56, 1.00)	1.59 (1.00, 2.52)	0.77 (0.45, 1.31)
**Older adults: 35+ years**							
	**Gender**						
		Female	Referent	Referent	Referent	Referent	Referent
		Male	0.88 (0.75, 1.03)	0.63 (0.53, 0.76)	0.70 (0.59, 0.82)	0.93 (0.74, 1.19)	0.63 (0.49, 0.83)
	**Race/ethnicity**						
		White, non-Hispanic	Referent	Referent	Referent	Referent	Referent
		African American, non-Hispanic	0.69 (0.53, 0.89)	0.95 (0.70, 1.29)	0.85 (0.66, 1.10)	0.71 (0.47, 1.09)	0.87 (0.58, 1.29)
		Hispanic	0.44 (0.32, 0.61)	0.39 (0.29, 0.53)	0.33 (0.24, 0.45)	1.28 (0.86, 1.91)	0.26 (0.12, 0.56)
		Other, non-Hispanic	0.96 (0.71, 1.29)	0.72 (0.51, 1.00)	0.72 (0.53, 0.97)	0.64 (0.39, 1.08)	0.69 (0.42, 1.14)

^a^A criterion of *P*<.01 was used to determine statistical significance.

^b^Site visits: 1 visit (registration visit) versus 2 or more visits.

^c^Time on site: 3 minutes or less versus more than 3 minutes.

^d^Page views: 11 pages or fewer versus 12 pages or more.

^e^Used Community: never versus any use.

^f^Used Separation Exercises: never versus any use.

## Discussion

### Principal Findings

This study compared utilization of a smoking cessation website among 18- to 24-year-old, 25- to 34-year-old, and 35 years and older smokers and examined differences by gender and race/ethnicity across age groups. Overall, both 18- to 24-year-old and 25- to 34-year-old young adults had lower levels of website engagement than older adults and were less likely to use specific features within the site. Within age groups, utilization patterns varied by gender and race/ethnicity. Women were more active on the site than men, but these gender differences were much larger in magnitude among young adults compared with adults aged 35 years and older. Minorities generally had lower levels of site utilization than white smokers and this was especially true for Hispanic smokers of any age. The exception to this pattern was use of the BecomeAnEx.org community: among 18- to 24-year-old and 25- to 34-year-old young adults, Hispanics were 2.4 to 3.5 times more likely than whites to use the BecomeAnEX.org community, whereas among older adults there were no differences in community use by race/ethnicity. There were few significant differences in utilization between the 18- to 24-year-old and 25- to 34-year-old subgroups by gender or race/ethnicity.

Among the few studies that have looked at website utilization differences between young and older adults, findings have been mixed [17,56-59]. Results from this study are somewhat counter to the Richardson et al [17] study, which found few differences in utilization between young and older adults. Differences between the findings from this study and the earlier study [17] may be due to confounding factors such as motivation to quit that were not available for the current analysis. A study by Sadasivam et al [57] found that young adult smokers were underrepresented on the Decide2quit.org website based on their representation in the general population. Another study examining Quitline callers who were offered a choice between a Web-only adjunct or a phone/Web adjunct found that Web-only participants were more likely to be younger [56]. However younger participants of either the Web-only or phone/Web option were less likely to return after the first visit [56]. Young adults have been described as hard to reach and engage with cessation treatment resources [10], and this may be true for online treatments as well.

Findings from a study by Klatt et al [60] as well as other research suggest that distinctive approaches may be needed to engage young adults, including education on how a Web-based program and other treatment modalities can improve cessation outcomes [44,58]. In addition, tailoring strategies may be needed to maintain young adult interest in website interventions. The website and associated promotional materials for this study were designed for a target audience of 25- to 49-year-olds; some of the messaging and imagery on the site may need to be adapted to resonate with a younger adult audience, particularly for the 18- to 24-year-olds. Young adults in this age range are in a period of life characterized by considerable change and instability—a phase of development in which individuals are exploring identities and seeking out a wide range of experiences prior to settling down [5]. Providing website interventions in a way that emphasizes exploration and highlights how cessation can enhance and broaden life experiences may help further engage young adult audiences versus focusing solely on the idea of quitting smoking. The research by An et al [27] which utilized an avatar host and conceptualized quitting smoking as a personal makeover suggests that such an approach may be both engaging and effective among younger adults. Despite lower utilization, it is noteworthy that over half of visitors during the 6-month period of this study were young adults—an encouraging signal that this is a viable channel to connect with this population.

Given the low utilization among young adult men, effective strategies to engage this population are critical. Health seeking is higher among women, which means that a smaller proportion of registered users are likely to be men in the first place [61]. Indeed, participants of Web-based cessation trials are more likely to be female [14]. Graham et al [62] found that banner ads targeted to men were effective in driving users to websites. However, studies have not generally focused on differences in engagement or efficacy of Web interventions by gender. Among men who seek out support online, an open question is how best to design Web-based cessation interventions that can sustain an initial level of engagement. Further, targeting online advertising to young male smokers to encourage engagement and reengagement with online cessation sites may be effective.

While young adults were less likely to use the BecomeAnEX.org community than older adults in adjusted analyses, the proportion of young adults utilizing the community was similar to that of older adults, primarily due to use by young adult Hispanics. The unusually high utilization of the BecomeAnEX.org community among Hispanic young adults may indicate an affinity for online group support for cessation among a population likely to be acculturated, native English speakers who are comfortable with online social networking. One key research question suggested by these findings is whether young adults prefer to interact with other people their same age or whether a community of current and former smokers of any age is appealing. Klatt et al [60] found that young adults who engaged with peers for online support were more likely to be abstinent at 30 days, which suggests that peer interaction may need to be an element of online cessation for young adults. Given the ubiquity of Web 2.0 tools, the daily use of social media among young adults [63], and the importance of peers and friendship networks for youth [64,65], an approach that incorporates social support and addresses social norms around smoking may resonate with this age group [23,40,45].

With the exception of use of the BecomeAnEX.org community, both young and older Hispanics were less likely than non-Hispanic whites to utilize the website. A previous evaluation of the website also found that Hispanics were less likely to visit the site compared with whites [17]. It is not clear why Hispanics were less likely to engage, because the site was designed for specific smoker populations including lower income groups and minorities, and a Spanish language version was available [66]. The high utilization of the BecomeAnEX.org community among both younger and older Hispanic adults suggests that incorporating additional options for sharing and social networking may be one strategy for maintaining engagement among the Hispanic population. Understanding how best to increase cessation website engagement for Hispanic smokers is key to ensuring effective treatment for a group that has the lowest rate of treatment utilization of all racial/ethnic groups [31].

Both the BecomeAnEX.org community and the Separation Exercises were significantly associated with quit outcomes in unweighted analyses in a previous evaluation study of the website [17]. The current study found that use of these tools was generally low across both young and older adults. Strategies to further highlight the most effective tools, including online communities, on cessation websites may be useful in increasing engagement overall and among low utilization subgroups. Use of active online community engagement during a quit attempt can help to increase cessation by providing social support [23] and steering new users to additional website features that are effective in helping smokers quit [23,47]. Given the rising use of and comfort with social networking activity in recent years among all demographic groups [32], seeking social support from online acquaintances who are going through similar experiences may become more popular for smokers attempting to quit.

### Limitations

Our ability to interpret these findings is constrained by the limited information we have about participants. Use of any tobacco cessation intervention is a function of a wide range of personal (eg, time, access) and psychosocial (eg, partner support for quitting, depression, self-efficacy for cessation) factors. Previous research in Internet smoking cessation interventions has found that changes in smoking temptations, quitting confidence, and positive and negative partner support mediated the relationship between treatment and abstinence and were strongly associated with increased website utilization [16]. Similarly, other research has found prior use of treatments and a belief that certain treatments are efficacious to be predictive of utilization [44]. Given that we did not have access to this kind of information about participants, it is not clear whether the observed age-related findings are truly a function of age or whether they reflect differences between younger and older adults on any of these unmeasured variables.

Further, utilization metrics presented here do not necessarily capture the full experience of a respondent on the site. For example, accessing the BecomeAnEX.org community does not capture differences in whether respondents simply viewed a page of the community or whether they posted or responded to a thread. Metrics such as time on site, pages viewed, or number of visits do not capture other aspects of engagement, such as how people are responding to the materials. Finally, site utilization may be shaped by website design factors including layout, website organization, and language, thus generalizability to websites with substantial design differences may be limited. While we acknowledge that these analyses are limited in their scope, this study is the first to our knowledge to document differential utilization patterns among age, gender, and race/ethnicity subgroups in an Internet cessation intervention. These findings point to important areas of inquiry to address in future research and development efforts.

This observational study was not designed to examine the links between website utilization and cessation outcomes. One meta-analysis of smoking cessation studies found that young adults were as likely to quit as older adults in studies with positive treatment outcomes [34]. However, given the nature of this observational study and the lack of cessation outcomes, we cannot conclude that lower levels of website utilization among young adults resulted in lower abstinence rates. It is possible that younger adults obtained the information and/or support they needed during their time on the site and this was sufficient in promoting some duration of abstinence.

### Conclusions

Further reductions in adult smoking rates require addressing tobacco use and cessation among young adults, a population that is increasingly vulnerable to long-term smoking addiction. Given young adults’ extensive use of online digital and social media, Web-based interventions are a promising intervention if younger smokers are adequately targeted and engaged. This study found overall lower levels of engagement among young adult subgroups ranging in age from 18 to 34 years compared with older adults, and patterns of use varied among age groups by race/ethnicity and gender. While findings are constrained by limited data, results suggest important areas of inquiry to address in future research and development efforts. Research focused on enhancing demand among younger smokers and men and providing effective features likely to be used once they reach a cessation site is key to helping users engage and stay motivated during a quit attempt. Incorporating features that capitalize on key aspects of young adult development, such as exploration of new experiences and the influence of peers, may also be effective in keeping young adults engaged. Identifying relevant and effective tools, including social networking apps and online community forums, to engage specific young adult subpopulations is critical to maximize the impact of Internet interventions, and this may be especially important for both young and older adult minority groups. Finally, strategies to increase utilization of effective site features among young adult users can help optimize cessation websites for an increasingly diverse user population.

## Appendix

Multimedia Appendix 1 Participant characteristics for ages 18-24 years, 25-34 years, and 35 years and older.

Multimedia Appendix 2 Bivariate analyses of website utilization metrics for ages 18-24 years, 25-34 years, and 35 years and older.

## Abbreviations

AOR: adjusted odds ratio
IQR: interquartile range
OR: odds ratio
PHS: Public Health Service
SE: standard error
