# Supplementary material for: Young Adult Utilization of a Smoking Cessation Website: An Observational Study Comparing Young and Older Adult Patterns of Use
Source: JMIR Res Protoc. 2016 Jul 11;5(3):e142. doi: 10.2196/resprot.4881 (PMC4960403; doi:10.2196/resprot.4881)
Supplement: Multimedia Appendix 1 [file resprot_v5i3e142_app1.pdf]

Multimedia Appendix 1: Participant characteristics for aged 18-24, 25-34 and 35+.

	**Young Adults 18-24 (n=957)**	**Young Adults 25-34 (n=2,073)**	**Older Adults 35+ (n=2,953)**	**Full Sample (n=5,983)**
Age, years, *Mean (SE)*	21.77 (0.06)	29.27 (0.06)	47.98 (0.16)	37.31 (0.16)
Gender, *N (%)*				
Female	539 (58.02)	1,028 (52.10)	1,998 (69.02)	3,565 (61.50)
Male	390 (41.98)	945 (47.90)	897 (30.98)	2,232 (38.50)
Race/ethnicity, *N (%)*				
White, non-Hispanic	570 (59.56)	1,183 (57.07)	2,268 (76.80)	4,021 (67.21)
AA, non-Hispanic	71 (7.42)	134 (6.46)	261 (8.84)	466 (7.79)
Hispanic	143 (14.94)	448 (21.61)	208 (7.04)	799 (13.35)
Other, non-Hispanic	173 (18.08)	308 (14.86)	216 (7.31)	697 (11.65)

Abbreviations: AA – African American; SE – standard error

Table 2. Bivariate analyses of website utilization metrics for aged 18-24, 25-34 and 35+.

	**Young Adults 18-24 (n=957)**	**Young Adults 25-34 (n=2,073)**	**Older Adults 35+ (n=2,953)**	**18-24 vs. 25-34 *P*-value**	**18-24 vs. 35+ *P*-value**	**25-34 vs. 35+ *P*-value**
Site visits						
Mean *(SE)*	2.32 (0.15)	2.61 (0.21)	7.59 (1.41)	.14	<.001	<.001
Median *(IQR)*	1.00 (1.00)	1.00 (1.00)	1.00 (2.00)			
1 time, *N (%)*	676 (70.64)	1,507 (72.70)	1,671 (56.59)			
2-3 times, *N (%)*	159 (16.61)	368 (17.75)	758 (25.67)			
4-8 times, *N (%)*	85 (8.88)	126 (6.08)	341 (11.55)			
> 8 times, *N (%)*	37 (3.87)	72 (3.47)	183 (6.20)			
Time on site, minutes;						
Mean *(SE)*	28.50 (3.91)	27.12 (3.04)	140.42 (32.84)	.003	<.001	<.001
Median *(IQR)*	5.95 (17.37)	4.52 (15.48)	10.98 (26.85)			
< 3 minutes, *N (%)*	345 (36.05)	887 (42.81)	713 (24.15)			
4-30 minutes, *N (%)*	449 (46.92)	881 (42.52)	1,497 (50.71)			
31+ minutes, *N (%)*	163 (17.03)	304 (14.67)	742 (25.14)			
Pages viewed;						
Mean *(SE)*	67.50 (15.63)	30.41 (3.32)	113.01 (23.61)	.001	<.001	<.001
Median *(IQR)*	10.00 (20.00)	8.00 (17.00)	14.00 (26.00)			
Used EX Community; *N(%)*	91 (9.51)	236 (11.38)	387 (13.11)	.12	.003	.07
Used Separation Exercises, *N (%)*	71 (7.42)	161 (7.77)	366 (12.39)	.738	<.001	<.001

Abbreviations: N – sample size; SE – standard error; IQR – interquartile range
